# Natural Compounds in Gastric Cancer Therapy: Molecular Mechanisms and Potential Treatment Options

**DOI:** 10.3390/ijms27020753

**Published:** 2026-01-12

**Authors:** Alexandra Dimaki, Lydia Lazaridou, Kalliopi Vakalou, Vasilios Zervas, Dimitra Bartzi, Kyriaki Tsagkidou, Panagiotis Dimitrios Papadopoulos, Konstantinos Eleftherios Koumarelas, Grigorios Christodoulidis

**Affiliations:** 1 Department of General Surgery, University Hospital of Larissa, 41110 Larissa, Greece; demakealexandra@gmail.com (A.D.); lazaridoulydia1@gmail.com (L.L.); kalliopi22003@gmail.com (K.V.); 2 Department of Internal Medicine, General Hospital of Athens “Evaggelismos”, Ypsilantou 45-47, 10076 Athens, Greece; vassilioszervas@gmail.com; 3 Department of Oncology, The 251 Airforce General Hospital, 11525 Athens, Greece; demmy345@hotmail.com; 4 Department of Gastroenterology, University Hospital of Larisa, 41100 Larisa, Greece; tsagkidoukyriaki@gmail.com; 5 Department of General Surgey, Spitalverbund Appenzel Ausserrhoden, Spital Herisau, Spitalstrasse 6, 9100 Herisau, Switzerland; panospapado1997@gmail.com (P.D.P.); kostaskoumarelas@gmail.com (K.E.K.)

**Keywords:** gastric cancer, curcumin, resveratrol, polyphenols, molecular mechanisms, anticancer therapy

## Abstract

Gastric cancer (GC) is the fifth most common type of cancer and a leading cause of cancer-related deaths worldwide. Surgery remains the most effective treatment, but new therapeutic strategies are urgently needed. The use of natural polyphenolic compounds such as curcumin (CUR) and resveratrol (RSV) has played a significant role in this effort. This review provides a comprehensive overview of the current applications and molecular mechanisms of curcumin and resveratrol in gastric cancer, highlighting their therapeutic potential and translational relevance. Analytically, CUR induces apoptosis, endoplasmic stress and cell cycle arrest. On the other hand, resveratrol enhances apoptosis and reduces inflammation. Both compounds increase cancer cell sensitivity to chemotherapy and help prevent chemoresistance, highlighting their potential as molecular enhancers in anticancer therapy. Combined with standard therapeutic drugs, they represent an innovative strategy for GC treatment. By presenting these innovative approaches, this review offers a global perspective on how their administration could shape future treatment strategies.

## 1. Introduction

Gastric cancer (GC) is classified as an aggressive disease with a poor prognosis, as most cases remain undetected until the subsequent phases when surgery and few chemotherapeutics become the sole recommended treatment method [1]. It is known as a multifactorial disease that is classified as the fifth most common cancer worldwide [2,3]. According to recent global estimates (GLOBOCAN 2024), GC accounts for approximately 970,000 new cases and over 770,000 deaths annually, making it the fourth leading cause of cancer-related mortality worldwide [4]. While incidence rates have declined in some Western countries, they remain alarmingly high in East Asia and certain regions of South America and Eastern Europe [4]. In the U.S., the age-adjusted annual incidence is 4.1 per 100,000, with a mortality of 1.6 per 100,000. Globally, the age-adjusted incidence is roughly double that, and the mortality rate is about four times higher [5]. The present situation has resulted in considerable attention being given to this tumor. Atypically, patients with gastric cancer have a poor prognosis due to tumor metastasis and regression. Consequently, the designation of a curative method is urgently needed [6]. Currently, treatment for GC patients is suboptimal because they are managed with standardized protocols, regardless of disease subtype. Recent advances in next-generation sequencing and genomic profiling have enabled in-depth characterization of the molecular heterogeneity of gastric cancers [7]. Nevertheless, in spite of the extensive amount of chemotherapeutic drugs that have been used, the overall survival of patients with GC has not improved significantly. Recent findings emphasize the natural environment as a key contributor to the discovery of novel antitumor agents. Natural polyphenolic compounds, especially curcumin (CUR) from *Curcuma longa* and resveratrol (RSV) from grapes, have shown strong anticancer effects in preclinical models of GC. Both compounds modulate critical signaling pathways such as PI3K/Akt/mTOR, NF-κB, Wnt/β-catenin, and Hedgehog, leading to apoptosis, inhibition of EMT, autophagy induction, and reduced cell proliferation and metastasis [8,9]. A number of studies have shown that plant-derived chemicals can suppress cancer cell proliferation and migration, stimulate apoptotic and autophagic cell decomposition, and increase the efficacy of chemotherapy [5].

**Figure 1:**
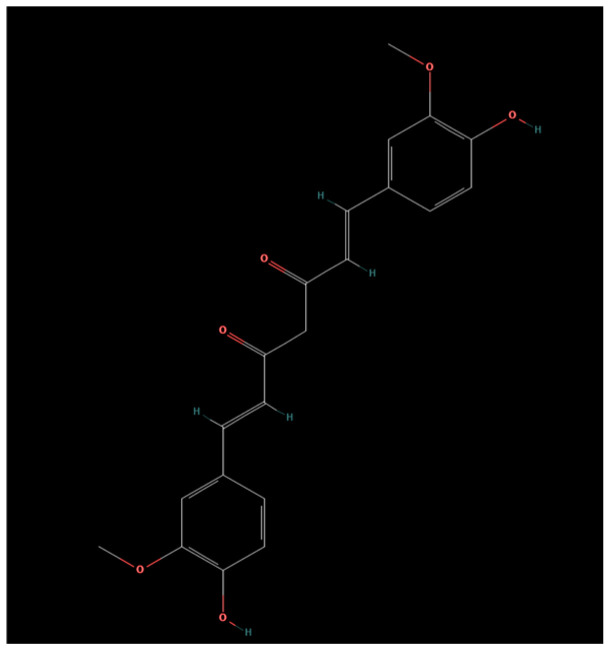
Chemical structure of curcumin [10].

Curcumin is a diarylheptanoid characterized by a symmetric structure comprising two aromatic rings substituted with phenolic and methoxy groups, linked by a seven-carbon heptadienedione chain that contains a conjugated β-diketone moiety. The correct IUPAC name most commonly used for curcumin is (1E,6E)(1E,6E)(1E,6E)-1,7-bis(4-hydroxy-3-methoxyphenyl)hepta-1,6-diene-3,5-dione [10] (Figure 1). Curcumin (CUR) is a natural polyphenol compound belonging to the Zingiberaceae family and is extracted from the dried rhizome of Curcuma longa L6 [11,12]. Studies have shown that it has anti-inflammatory and anti-oxidative effects and is also widely recognized as a strong antitumor agent [13,14]. Furthermore, it has a strong impact on inducing apoptosis and autophagy in tumor cells and regulating the cell cycle, resulting in the inhibition of metastasis and contributing to its chemo preventive and therapeutic effect [13]. It has been proven that CUR does not cause any toxicity despite being consumed for years in Asian countries. The main obstacle to using CUR for anti-cancer treatment is its low oral bioavailability due to limitations of poor water solubility and rapid metabolism [12].

**Figure 2:**
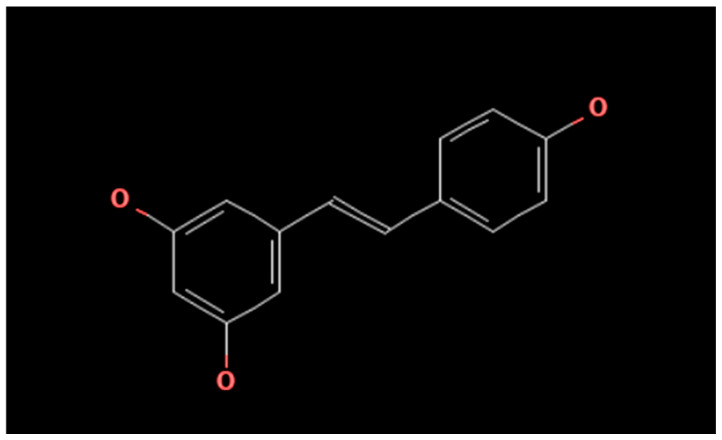
Chemical structure of resveratrol [15].

Resveratrol is a polyphenolic stilbene derivative consisting of two benzene rings connected by an ethene bridge in a trans configuration. Its IUPAC name is 3,5,4′-trihydroxystilbene, reflecting hydroxyl substituents at the 3 and 5 positions of one phenyl ring and the 4′ position of the other ring. This substitution pattern and conjugated −CH=CH− linkage contribute to its antioxidant and biological activity profile [15] (Figure 2). Resveratrol (RSV) is a polyphenol found in grapes, berries, peanuts and red wine; in either case, CUR and RSV share the same antioxidant, anti-inflammatory, and antitumor effects [16]. Mechanisms of RSV include regulation of the cell cycle, cancer cell apoptosis, and inhibition of epithelial–mesenchymal transition, resulting in cells becoming sensitive to chemotherapy [17]. Despite their potential, both CUR and RSV face substantial limitations in clinical application due to pharmacokinetic challenges, including low bioavailability, rapid metabolic degradation, and limited systemic distribution. Novel drug delivery systems—such as nanoparticles, liposomes, photodynamic formulations, and structural analogs—have emerged to improve stability, targeting, and efficacy [18,19,20]. Furthermore, recent clinical studies have begun to explore the safety, optimal dosing, and therapeutic synergy of these compounds with existing chemotherapy regimens. However, robust clinical validation is still lacking [17,21]. Among the natural substances that have been studied for gastric cancer (GC), curcumin (CUR) and resveratrol (RSV) stand out for their many and diverse molecular actions. Also noteworthy is their potential to enhance established therapy. Both compounds act on specific pathways related to cell survival and metastasis. These include PI3K-AKT-mTOR, NF-κB, MAPK, Hedgehog, and Wnt/β-catenin. As a result, inhibition of cell proliferation, induction of apoptosis, and reversal of epithelial–mesenchymal transition (EMT) are observed. CUR inhibits the PI3K-AKT-mTOR pathway. Thus, it induces apoptosis, autophagy, and, according to more recent studies, ferroptosis [8]. At the same time, it prevents the activation of NF-κB since it stabilizes IκBα while reducing the phosphorylation of p65. This leads to a decrease in the expression of COX-2, IL-6 and MMPs [22]. In combination with chemotherapeutics such as 5-fluorouracil (5-FU) and cisplatin, CUR increases cytotoxicity and overcomes chemoresistance mechanisms [22]. On the other hand, RSV restores GSK3β activity in the PI3K-AKT-GSK3β-Snail pathway. It also destabilizes the Snail protein and reverses EMT [17]. Additionally, it inhibits the Raf/MAPK pathway and limits IL-6-induced metastasis [17]. The complementary actions of CUR and RSV have highlighted their potential as adjuvant therapy candidates. They are also capable of regulating multiple oncogenic networks while enhancing the action of established chemotherapy.

This review aims to compile and assess the current evidence regarding the anticancer effects of resveratrol and curcumin in gastric cancer. It examines their molecular mechanisms of action, their ability to modulate autophagy, apoptosis, and metastasis, and their potential to enhance the efficacy of standard chemotherapy. Additionally, we explore delivery strategies, pharmacokinetic obstacles, and insights from recent clinical trials, aiming to present a translational perspective for integrating these compounds into future GC therapies.

## 2. Materials and Methods

This pooled analysis was conducted in accordance with a modified PRISMA (Preferred Reporting Items for Systematic Reviews and Meta-Analyses) framework, adapted for case reports and case series. A comprehensive literature search was conducted using six major databases: PubMed, Scopus, Web of Science, MEDLINE, Cochrane Library, and the ClinicalTrials.gov registry, covering the period from 2019 to the present day. The primary objective was to identify relevant publications exploring the role of natural polyphenolic compounds, particularly curcumin and resveratrol, in the context of gastric cancer treatment and prevention.

The search strategy employed a combination of Medical Subject Headings (MeSH) and free-text terms, using logical operators “AND” and “OR”. The specific search terms included the following: “Curcumin”, “Resveratrol”, “Stomach Cancer”, “Gastric Cancer”, “Gastric adenocarcinoma”, and “Gastric neoplasm”. Studies published in languages other than English, duplicate records, and conference abstracts without full data were excluded from the final pool.

Inclusion criteria were defined as follows:

(i) Original articles, systematic reviews, or meta-analyses focused on the use of curcumin and/or resveratrol in in vitro, in vivo, or clinical models of gastric cancer;
(ii) Studies elucidating molecular mechanisms, such as regulation of apoptosis, oxidative stress, or cell signaling pathways.
(iii) Reports investigating synergistic effects with conventional chemotherapeutic agents or novel delivery formulations.

**Figure 3:**
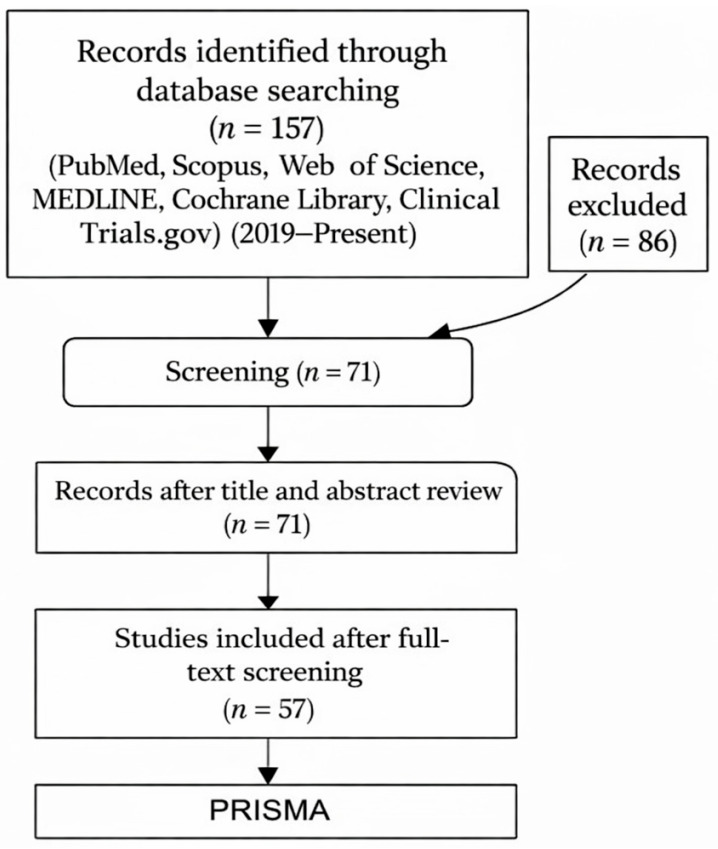
Molecular Mechanisms of Polyphenols.

The initial search yielded 157 studies from 2019 to present. Following title and abstract screening, 71 articles were retrieved for full-text assessment. After detailed evaluation, 57 studies were considered eligible and were included in the final review (Figure 3).

Curcumin (CUR) and Resveratrol (RSV) exhibit a variety of anticancer properties by modifying interrelated molecular pathways essential to gastric carcinogenesis, whether or not they are shared. Through epigenetic reprogramming (lncRNA/miRNA networks and histone/DNA modifications) and dysregulation of important signaling cascades (e.g., PI3K/AKT/mTOR, Wnt/β-catenin, and NF-κB, Hh), both substances promote intrinsic and extrinsic apoptosis, alter redox equilibrium, impede cell cycle advancement (G1/S and G2/M arrest), and prevent the spread of metastases. Their distinct yet complementary polypharmacology, which consists of the upstream pathway modulation of RSV and the direct multi-target engagement of CUR, explained mechanistically below, places them in a strong position as chemointerceptive agents that provide a multifaceted therapeutic approach against gastric cancer and oncological applications [Table 1].

### 2.1. Molecular Mechanisms of Curcumin

Curcumin (CUR), the principal bioactive polyphenol derived from Curcuma longa rhizomes, orchestrates an outstanding pleiotropic anticancer program through its ability to simultaneously modulate more than 60 distinct molecular targets across multiple cellular compartments, signaling hierarchies, and biochemical interactions. This systems-level bioactivity arises from curcumin’s unique chemical structure, which is characterized by a symmetrical β-diketone flanked by phenolic rings, enabling both covalent and non-covalent interactions with transcription factors, kinases, receptors, growth factors, inflammatory cytokines, and epigenetic regulators. At the apoptotic level, curcumin engages both extrinsic (death receptor) and intrinsic (mitochondrial) pathways with remarkable precision. It activates caspase-8 via Fas/FasL clustering [8], while concurrently inducing mitochondrial outer membrane permeabilization (MOMP) through Bax oligomerization, Bak activation, and Bcl-2/Bcl-xL downregulation [11,22]. This dual engagement converges on the activation of caspase-3/7 and PARP cleavage. Critically, CUR enhances apoptotic signaling through endoplasmic reticulum (ER) stress. This is accomplished by inhibiting SERCA2 ATPase to deplete ER calcium stores, thereby triggering the unfolded protein response (UPR) with sustained PERK/eIF2α/ATF4/CHOP axis activation [19,23]. Thus, the transcription of CHOP represses Bcl-2 while upregulating Bim and DR5, creating a feed-forward apoptotic loop [8].

The substance’s capacity to disrupt the cell cycle is performed through multi-checkpoint interference at G1/S and G2/M phases. At the G1/S transition, CUR suppresses cyclin D1-CDK4/6 complex formation by inhibiting NF-κB and AP-1, thereby repressing the transcription of CCND. This leads to the activation of ROS-dependent E3 ubiquitin ligase, which causes the proteasomal degradation of CDK4 and degrades CDK4 mRNA in a miR-34-dependent manner, resulting in epigenetic silencing [8,9,23]. For G2/M arrest, CUR inactivates CDC25C phosphatase through JNK-mediated phosphorylation, preventing CDK1 dephosphorylation and the activation of the cyclin B1-CDK1 complex, consequently effectively blocking mitotic entry [9,24].

In the suppression of oncogenic pathways, curcumin exhibits masterful modulation of crosstalk. Within the Wnt/β-catenin cascade, it binds to Frizzled receptors to block Wnt3a docking, phosphorylates β-catenin at Ser45 via CK1α, priming it for GSK3β-mediated degradation, and disrupts β-catenin/TCF4 nuclear complexes via competitive binding to TCF’s DNA-interaction domain [8,22,23]. The PI3K/Akt/mTOR axis is crippled through PTEN reactivation, mediated by miR-21 downregulation and direct inhibition of PI3K’s p110 catalytic subunit. This suppresses PDPK1-mediated Akt phosphorylation at Thr308, in turn destabilizing mTORC1 and blocking S6K1/4E-BP1 activation [8,9,11,25]. NF-κB inhibition involves the inactivation of IKKβ through cysteine-179 alkylation, the stabilization of IκBα via proteasome inhibition, and the nuclear exclusion of p65 through impaired importin-α3 binding [8,22,26].

CUR exhibits a multi-target mechanism for inhibiting oncogenic signaling in GC, primarily affecting the PI3K/AKT/mTOR, EGFR/Src, NF-κB, STAT3, and Wnt/β-catenin pathways. This action negatively regulates the transcription of factors involved in epithelial–mesenchymal transition (EMT), as well as genes for matrix metalloproteinases and survival and anti-apoptotic proteins [16,21,27]. Experimental evidence used in vitro and in vivo systems clearly indicates that curcumin negatively regulates vascular endothelial growth factor (VEGF), VEGF-D, and HMGB1 proteins to repress angiogenesis and lymphangiogenesis. This also includes a reduction in GC-MSC-induced tube formation and endothelial cell migration through NF-κB/VEGF downregulation. At the epigenetics level, curcumin targets numerous cancer-related microRNAs, such as miR-21 and its target genes (PTEN and PDCD4), along with other cancer-related miRNAs [6,14,21,28]. This re-expresses tumor-suppressive pathways with a concomitant reduction in proliferation and tumor development, as well as increased sensitization toward apoptotic cell death. This particular procedure for modifying miRNAs also includes changes in histones and DNA methylation levels. CUR displays a context-dependent pro-oxidant action in cancer cells with increased reactive oxygen species (ROS) levels, leading to mitochondrial depolarization, cytochrome c release, activation of caspase 9 and 3, Bax/Bak upregulation, and Bcl-2 downregulation to regulate intrinsic pathway activation [29,30,31]. CUR also arrests cell cycle progression at both G1/S and G2/M phases with concomitant downregulation of cyclins and cyclin-dependent kinase (CDKs) and subsequent upregulation of p53, p21, and p27 [29,30,32,33]. This action may limit tumor growth mediated by clones with a potential for progression and establishment of micrometastasis. CUR may also inhibit activation and resultant pro-angiogenic properties of mesenchymal stromal cells and fibroblasts of gastric cancer origin. Alpha-SMA and vimentin may be downregulated. CUR may modulate NF-κB-dependent signaling for vascular endothelial growth factor (VEGF) between stromal and cancer cells. CUR also targets a multitude of pathways associated with redox regulation, cell cycle progression, apoptosis regulation, and crosstalk between stromal and cancer cells. It acts as a broad-spectrum multi-target drug with potential for attenuation of epithelial–mesenchymal transition, along with invasion and survival events associated with progression throughout a cascade leading to the establishment of micrometastasis [9,29,32].

Epigenetic reprogramming represents a fundamental mechanism. CUR not only inhibits DNMT1 by covalent binding to Cys1226 in the catalytic domain, inducing global hypomethylation, but also suppresses p300/CBP HAT activity (CUR concentration = 25 μM), resulting in a reduction in H3K27ac at oncogene promoters. Furthermore, CUR plays a crucial role in modulating lncRNA/miRNA networks by upregulating miR-34a through p53 activation, while degrading MALAT1 via enhanced EXOSC5-mediated nuclear export [8,9,23,34].

The redox duality of CUR is primarily dependent on the substance’s concentration. At concentrations <10 μM, Cur activates Nrf2/ARE signaling through Keap1 cysteine modification (Cys151/Cys288), inducing HO-1, NQO1, and GSTs to mitigate oxidative stress [26,35]. At concentrations greater than 20 μM, it promotes the generation of ROS/RNS via the inhibition of mitochondrial complex I/III activation of xanthine oxidase, and induction of iNOS. Consequently, GPX4 inactivation and ACSL4-mediated lipid peroxidation lead to ferroptosis [9,24]. Within the tumor microenvironment, CUR is closely related to the normalization of aberrant angiogenesis. This is derived from its ability to block HIF-1α nuclear translocation under hypoxia, inhibit VEGFR2 tyrosine kinase activity, and successfully suppress the secretion of SDF-1/CXCL12 by cancer-associated fibroblasts (CAF) [8,9,22].

In line with the broader polyphenol class, curcumin exerts direct molecular effects on enzymes critical for genomic maintenance, including DNA topoisomerases and DNA polymerases, thereby interfering with DNA replication, transcriptional fidelity, and DNA damage repair in rapidly proliferating gastric cancer cells [30]. In tumors, curcumin downregulates repair polymerases (e.g., Polβ and FEN1) and related proteins, blocking BER and homologous recombination to sensitize cells to DNA damage and PARP inhibitors. High doses trigger ROS-mediated damage, reducing polymerase efficiency via p53 activation and DNMT1 repression, leading to apoptosis [36]. Curcumin derivatives bind the BRCT domain of human DNA polymerase λ, potentially modulating its activity, though replicative polymerases (α, δ, ε, γ) remain unaffected [37].

CUR can act as a “topoisomerase poison,” stabilizing topoisomerase–DNA cleavage complexes (Topoisomerase I and Topoisomerase II) in some cancer cell lines, which contributes to DNA damage and antiproliferative effects [38].

In addition, the compound also exhibits anti-H.pylori capabilities: Curcumin cagPAI gene cluster repression via NikR inhibition, VacA hexamerization blockade by binding to the p55 intermediate domain, and urease inhibition through competitive binding to the active-site nickel center [26,39].

Chemosensitization is attributed to the substance by exploiting the thermodynamics of the ABC transporter. CUR binds MDR1’s nucleotide-binding domain with ΔG = −9.8 kcal/mol, impeding ATP hydrolysis and drug efflux [9,22,26]. Mitochondrial sabotage encompasses mtDNA depletion via POLG inhibition, disruption of the ANT-VDAC complex, leading to mitochondrial membrane potential collapse, and inhibition of the mitoKATP channel, thereby preventing cytoprotective volume regulation [22,25]. Its chemopreventive role includes CYP1A1 inhibition to block BaP-induced DNA adducts and p53 reactivation in mutant backgrounds, triggering the DNA-binding domain to refold [40]. This comprehensive, multi-omic targeting spanning genomic, transcriptomic, proteomic, and metabolomic axes positions curcumin as a paradigm-shifting polypharmacologic agent with unmatched mechanistic breadth against gastric cancer and related oncological applications.

### 2.2. Molecular Mechanisms of Resveratrol

Resveratrol (RSV), a natural polyphenol found in grapes and berries, has garnered significant attention for its multifaceted anticancer properties, particularly its ability to regulate apoptosis, inhibit proliferation, and disrupt key signaling pathways involved in gastric cancer progression. The Bcl-2 protein has a major effect in stopping apoptosis, among which is an antiapoptotic protein comparable to Bcl-2 and B-cell lymphoma-extra broad (Bcl-xl), along with a proapoptotic protein identical to Bcl-2 associated X (Bax) and Bcl-2 associated Leader of Cell Death [9]. RSV may induce mitochondrial apoptosis, either Bax-dependent or Bax-independent, via the intrinsic or mitochondrial nerve pathway. RSV promotes Bax protein colocalization to the mitochondrion, collapse of the mitochondrial membrane, activation of caspase 3 and 9, and finally, apoptosis. Also, it causes a rapid increase in mitochondrial ROS production prior to caspase activation [41]. Exposure of GC cells among RSV increases the cell’s morphological features of apoptosis, similar to chromatin condensation, chromatin crescent formation, and nucleus atomization [9,16]. RSV activates Fas receptor redistribution in membrane tons to trigger apoptosis in the extrinsic nerve pathway. The polyphenol prompts cluster and reposition of Fas, which is related to death signals complexed with cholesterol and sphingolipid fractions of SW480 cells, in conjunction with Fas-binding protein with cell demise area and procaspase-8. Lysosomes and the endoplasmic reticulum can also initiate apoptosis [41]. Moreover, ROS-triggered autophagy is a mechanism through which RSV induces apoptosis. This can be partially inhibited by the PKC-ERK1/2 signaling pathway. RSV significantly increases PKC and ERK1/2 phosphorylation. Apoptosis is promoted by pretreatment with PKC and ERK1/2 inhibitors G6976 and PD98059, respectively [41]. RSV-stimulated autophagy provokes essential suppression of apoptosis, associated with diminished cleavage efficiency of caspase-8 and caspase-3. Administration of RSV significantly increases the intracellular level of dihydroceramide (Des1 and Des2) to trigger autophagy, a major factor affecting cell viability and proliferation, and sensitizes these malignant cells to apoptosis [16].

RSV has anti-tumor and anti-metastatic effects in GC, primarily via reprogramming of the receptor–cytokine–transcription factor axis without direct inhibition of kinase hubs. It targets several pathways, such as TGF-beta-SMAD, Hedgehog, Hippo-YAP, and NF-κB-STAT3, leading to inhibition of epithelial–mesenchymal transition (EMT) via downregulation of N-cadherin and vimentin and concomitant upregulation of E-cadherin levels, along with a subsequent decrease in the migration and invasion abilities of GC cells [16,32,42]. RSV also targets IL-6 mediated pro-survival and pro-invasive signaling; this has shown attenuation of IL-6-induced invasion with the potential capability of blocking Raf/MAPK and NF-κB activation mediated by IL-6 invasion [16,43,44]. RSV epigenetically targets a number of miRNAs, such as downregulation of oncogenic miR-21, along with regulation of other genes, such as MALAT1 and other Non-Coding-RNAS involved in EMT and invasion, with a subsequent increase in levels of tumor-suppressive target genes such as PDCD4.

Similar to CUR, RSV may also increase intracellular reactive oxygen species levels in cancer cells in a specific manner for a particular dose range, thereby linking oxidative stress with mitochondrial damage, release of cytochrome c, and activation of caspases. Additionally, RSV causes cell cycle arrest in G1 and G2/M phases of cancerous cells using downregulation of cyclin proteins and cyclin-dependent kinase proteins and via p53 signaling pathways for a reduced clonogenic survival fraction with increased responsiveness to chemotherapy drugs such as 5′-Fluorouracil and cisplatin [29,30,32,33]. Also, in cancerous environments, NF-κB-induced pro-inflammatory cytokines and chemokines, such as TGFβ and IL6 levels, are downregulated [16,44]. This leads to decreased crosstalk interactions between cancer cells and cancer-associated stromal fibroblasts and tumor-associated macrophages. This typically leads to inhibition of EMT, angiogenesis, and evasion of the immune response. Thus, primarily, RSV operates as a transcription factor modulator and a cytokine receptor signaling modulator. This approach complements CUR’s strong action as a kinase receptor and epigenetics-modulator with a strong focus toward addressing a combinational inhibition of EMT, invasion, angiogenesis, survival signaling pathways, and redox signaling pathways [21,27,30].

Apoptosis is one of the most important mechanisms causing tumor cell death and a key step in anticancer therapy. Studies indicate that RSV blocks the cell cycle in the S phase and induces cell apoptosis to suppress AGS cells. In vitro experiments have shown that RSV inhibits AGS cell proliferation, migration, and invasion and induces S phase apprehension and apoptosis by targeting FOS and MMP9 [17]. Moreover, RSV inhibits excessive epithelial cell proliferation by changing the protein that enters the cell cycle and prefers cyclins and CDKs via the regulated PI3K/AKT (PTEN), TGF-, and Smad signal nerve pathway [16]. The combined statistics reveal that RSV can also manipulate several signal nerve pathways in the treatment of irregularities. The downregulation of protein kinase C (PKC) by RSV may be related to cell viability and growth. It has been demonstrated that PKC has anti-proliferative and pro-apoptotic effects. RSV administration increases PKC cytosol release and decreases membrane PKC protein. Such impacts lead to increasing p21 and p53. In addition, RSV treatment facilitates the Fas and Fas-L protein stages. These findings are entirely due to the stimulation of cell cycle arrest in the G2/M phase and the killing of apoptotic cells to suppress GC malignancies. RSV chemotherapeutic activities mainly depend on its influence on PKC. The development of indications indicates that PKC participates in the progression of tumors, tumor proliferation, viability, and migration of tumors. RSV has a negligible effect on cell lysis, decreasing the expression of a related protein in the signal nerve pathway identical to cyclin D1, which is essential for the G0/G1 cell cycle [16,41]. The PI3K/AKT signal nerve pathway may remain abnormally activated, mainly in cancers, while PTEN regulates a number of cell signal nerve pathways [17]. PTEN upregulation may therefore inhibit the PI3K/AKT/mTOR pathway [40]. The PI3K/AKT/mTOR nerve pathway participates in a variety of cell biological processes, such as reducing glycolysis [45]. The abnormal activation of the Wnt/β-catenin signal nerve pathway is one of the major mechanisms of human tumorigenesis, and β-catenin overexpression is a major manifestation of the activation of the current signal nerve pathway. While the current nerve pathway is not functioning properly, unphosphorylated or undegraded β-catenin enters the nucleus and adheres to the linked field of transcription factor (TCF) -4 toward the structure of the transcribed region. Then, it mixes with related transcription components to create a downstream target gene and support tumorigenesis [46,47]. MALAT1 is also correlated with the Wnt/β-catenin signal nerve pathway, which governs tumor cell invasion and metastasis. In addition to reducing the nuclear localization of β-catenin, treatment with RSV may result in a reduction in the Wnt/β-catenin signal [41]. Another important event related to tumorigenesis is the activation of the nuclear factor kappa B (NF-κB) gesture transduction nerve pathway. RSV has significant results against H. Pylori-induced oxidative stress and inflammation by suppressing IL-8 and iNOS expression, barricading NF-κB activation, and disrupting the nuclear factor erythroid 2-related factor 2 (NRf2)/heme oxygenase-1 nerve pathway [9,41]. The molecular signal nerve pathway demonstrated that IL-6 promotes cancer cell progression by initiating the Raf-MAPK signaling pathway. Similarly, the administration of RSV suppresses IL-6-mediated invasion of GC via the inhibition of the Raf-MAPK signal nerve pathway [16,20,46]. In particular, the Hh nerve pathway stimulates EMT in GC. Therefore, the transition of this signal nerve pathway is important for the suppression of migration and metastasis of cancer cells. It appears that Gli-1 is a biomarker of Hh nerve pathway abnormal expression. RSV administration significantly disrupts the Hh nerve pathway by downregulating Gli-1 [16]. As a result, Snail and N-cadherin, together with E-cadherin, suppress EMT to reduce invasion and migration of GC cells. The detection of EMT markers E-cadherin, N-cadherin, Vimentin, and Snail demonstrated that E-cadherin had reduced messenger RNA and protein expression, while the messenger RNA and protein expression of Vimentin, N-cadherin, and Snail increased in the TGF-1 group compared to the control group [41,48]. Moreover, MALAT1 lncRNA downregulation by RSV inhibits proliferation, migration and invasion of human cells in gastric cancer [16,49]. Yang et al. [50] found that a decrease in DDIT4 and miR-383-5p expression leads to a decrease in the anticancer activity of miR-383-5p, which suggests that miR-383-5p may have an anticancer effect by targeting DDIT4 and regulating DDIT4 expression. Thus, the results obtained so far indicate that the MALAT1/miR-383-5p/DDIT4 nerve pathway may be a potential nerve pathway in the treatment of a GC patient. The consequences of RSV on gastric cancer refer to the control of miR-155-5p, whose overexpression contributes to oncogenesis. The results demonstrated that miR-155-5p expression decreased with RSV therapy in a dose-dependent manner [8]. Increased SOD activity in a dose-dependent manner was observed with RSV treatment; the increase was statistically significant only when high concentrations of the substance were used [9,51]. RSV is a non-poison topoisomerase II inhibitor that binds at the ATPase dimerization interface and inhibits the ATP-dependent strand-passage activity of topoisomerase II, thereby blocking DNA relaxation and decatenation without stabilizing DNA breaks [52]. RSV potently inhibits DNA polymerases α and δ (replicative enzymes), with IC50 values correlating with its antiproliferative activity; this occurs via binding through its 4′-hydroxystyryl moiety, blocking DNA synthesis progression. It also suppresses viral DNA polymerases, such as in poxvirus replication, by interfering with DNA synthesis steps post-early gene expression [53]. In addition, RSV is reported to suppress urease production and cause a decrease in the gut niche’s pH, resulting in the death of H. pylori due to its high acidity. Finally, the polyphenol regulates the protein expression of various toxic factors (CagA) responsible for H. pylori-induced gastric cancer [46].

## 3. Polyphenols and Therapy

Natural polyphenols, especially CUR and RSV, appear to be an adjuvant treatment for stomach cancer. In particular, they help existing anticancer therapies, covering gaps in classical chemotherapy through various multifactorial mechanisms. Thus, they enhance the effectiveness of chemotherapy while at the same time overcoming resistance to various cancer drugs.

Initially, it was shown that the action of many chemotherapeutic drugs, such as 5-fluorouracil (5-FU), cisplatin, doxorubicin (DOX), and vincristine (VCR), is enhanced by CUR when administered concomitantly. Specifically, they act by reducing cell growth at a rate of up to 93%. This is achieved by inhibiting P-glycoprotein, suppressing the signaling of the NF-κB pathway and activating caspase-3 [8,21]. In addition, when CUR is combined with 5-FU and cisplatin, it is observed that cell viability and colony formation are reduced, apoptosis is induced, and migration is blocked through the regulation of Bcl-2, Bax and -3/-8 proteins. In particular, CUR increased caspase-3/-8 activity, downregulated Bcl-2 and upregulated Bax [8]. In models that feature multi-resistance (MDR), such as SGC7901/VCR, CUR reduces the IC50 of vincristine while reversing treatment resistance. Furthermore, when combined with PD98059 (MAPKK inhibitor), it promotes apoptosis in MGC-803 GC cells, as the MAPKK pathway regulates proliferation, transcription, differentiation, survival, and apoptosis [8], while when combined with doxorubicin (DOX), it leads to increased apoptosis, decreased migration and cell invasion [54]. Through studies conducted in the UK, in phases I/IIa, it was observed that the addition of CUR to the FOLFOX regimen has anticancer activity and is safe and potentially beneficial [21], while in mice and after administration at low doses (160 mg/kg/day), it significantly reduced lymphatic vessel density (LVD), a key marker of lymphatic metastasis in stomach cancer [25]. Even when administered in a smaller amount (10 mg/kg/day), in combination with 5-FU and oxaliplatin, it was shown that it can work cooperatively with chemotherapy and induce apoptosis of cancer cells [25]. In a clinical trial for gastritis associated with Helicobacter pylori, standard triple therapy and triple CUR supplementation were compared. In the CUR group, increased total antioxidant capacity in the gastric mucosa was found, oxidative stress markers and the rate of eradication of Helicobacter pylori were improved, and oxidative damage to DNA was reduced [35].

CUR mechanically promotes iron deficiency in gastric cancer (GC) cells. In particular, intracellular iron levels increase significantly after CUR treatment, as do malondialdehyde (MDA) and ROS levels, while glutathione (GSH) levels decrease. This is achieved by altering the way the protein to which ferrofall is associated changes. That is, there is increased regulation of ACSL4 (promoter of lipid peroxidation in cripple) and reduced regulation of SLC7A11 and GPX4 (key cripple suppressants) [34].

In addition, the action of CUR can also be enhanced through photodynamic therapy. CUR is a photosensitive photosensitizer (PS). It is activated by light to produce reactive oxygen species (ROS). Through its photodynamic action, it selectively targets cancer cells without harming normal cells. Thus, it becomes a very safe treatment option [19]. Photodynamic therapy (PDT) using CUoffers a minimally invasive strategy with targeted ROS generation in malignant tissue [34].

Similarly, RSV enhances apoptosis induced by cisplatin and promotes cell cycle arrest in the G2/M phase, while at non-toxic concentrations (5–12.5 μM) it inhibits metastasis through downregulation of MALAT1 lncRNA [9,50].

Specifically, in AGS cells, when RSV is administered in combination with cisplatin (DDP), the activity of beta-galactosidase (an indicator of cellular aging) increases, while ROS levels, which indicate oxidative stress, also increase. At the same time, it was observed that there is disruption of the cell cycle in the G0/G1 phases as well as increased regulation of p53, p38, p16, p21, and MMP-2. In this way, the apoptosis of cancer cells is thus enhanced [9,55]. It was also observed that the combination of RSV and cisplatin also caused cell cycle disruption in the G2/M phase. However, RSV was also administered with copper in a clinical trial with a phase II arm (October 2019–April 2021). The results of this study showed that when administered together they reduce the toxicity of docetaxel-based multiagent chemotherapy. Finally, improved tolerance to chemotherapy was observed in patients [17]. In addition, RSV activates PTEN to inhibit drug resistance induced by epithelial–mesenchymal transition (EMT). Activation of PTEN leads to suppression of Akt signaling. Thus, the sensitivity to the drug is enhanced. Also, RSV may reduce resistance to Doxorubicin (DOX) by stimulating PTEN resistance and suppressing EMT [16]. RSV treatment suppresses MALAT1 and cell proliferation. It was observed that RSV at increasing concentrations (up to 5 μM) decreased the expression of MALAT1 (lncRNA involved in cancer progression). However, both RSV and si-MALAT1 individually inhibited migration and invasion. However, it was noteworthy that the highest rates of apoptosis occurred under combined RSV + si-RNA therapy [50].

Both compounds also affect redox homeostasis: CUR induces ferroptosis through increased expression of ACSL4 and inhibition of GPX4 [34], while RSV reduces oxidative stress by increasing levels of antioxidant enzymes such as SOD and CAT and suppressing LPS-induced inflammatory activation of NF-κB [41,56].

RSV also reduces inflammatory cytokine production, including IL-2, IFN-γ (in lymphocytes), and TNF-α and IL-12 (in macrophages), with additional dose-dependent suppression of IL-1α, IL-6, and TNF-α. Moreover, its precursor, polydatin, displays similar anti-inflammatory activity, decreasing IL-6, IL-1β, and TNF-α levels in Mycoplasma gallisepticum infections, while boosting antioxidant enzyme levels (SOD, CAT, GPX), thereby reducing oxidative damage. Today, RSV is administered in new forms, such as RES@ZIF-90 (RSV in imidazole zeolite frameworks) nanoparticles. In particular, this route of administration demonstrated superior tumor inhibition in models of HGC-27 xenografts compared to free RSV. Thus, it was observed that the efficacy of tumor administration and targeting was improved [9].

Likewise, curcumin microsponge formulations have demonstrated up to a 10-fold increase in bioavailability, addressing its rapid metabolic degradation and poor water solubility. Despite promising effects, limitations in bioavailability remain a major challenge. Both CUR and RSV undergo rapid metabolic breakdown and have low water solubility, which hinders their therapeutic applications. Innovative drug delivery systems, such as CUR microsponges (10× increase in bioavailability) and RSV@ZIF-90 nanoparticles, improve tumor targeting and allow controlled release [9,19].

The future of polyphenol-based therapies lies in personalized, biomarker-guided approaches. For example, oxidized derivatives of CUR (such as cyclobutyl-cyclopentadione) show stronger inhibition of the Helicobacter pylori VacA protein than natural CUR, suggesting that structural optimization could enhance effectiveness [39].

Similarly, RSV exhibits dose-dependent action—cytotoxic at high concentrations (25+ μM) but anti-metastatic at lower doses—highlighting the need for precise dosage control [51]. Large-scale clinical trials are critical to confirm these findings, especially in combination strategies (such as CUR + FOLFOX in colorectal cancer) [21] or in synergy with immunotherapies. By leveraging advanced delivery systems and exploring mechanisms like epigenetic regulation (e.g., HDAC inhibition and MALAT1 targeting), polyphenols may redefine cancer treatment as low-toxicity, multitarget agents capable of overcoming drug resistance and improving patient outcomes.

## 4. Discussion

Curcumin (CUR) and resveratrol (RSV) exhibit significant therapeutic potential. They can also be utilized as adjuvant drugs in combating gastric cancer. This is accomplished through their multifactorial safety characteristics. Specifically, it was discovered that the administration of CUR up to 8 g/day results in no toxicity. Their antitumor effects are mediated through modulation of multiple signaling pathways, including PI3K/Akt/mTOR, Wnt/β-catenin, and NF-κB, as detailed above. In addition, they improve the efficacy of many chemotherapeutic drugs [8,11]. However, their therapeutic use is limited due to poor bioavailability. Poor water solubility (~11 ng/mL) is one of the key limitations. It limits bioavailability and tissue distribution. Moreover, rapid phase II metabolism in the liver and intestine results in the generation of inactive metabolites. Pharmacokinetic constraints entail possessing a poor half-life and rapid systemic clearance by the liver and kidneys [12]. To reduce this limitation, new routes of delivery have been developed. These include advanced formulations such as nanoparticles and liposomes. These nano-delivery systems address clinical bottlenecks by encapsulating the hydrophobic molecules within a protective shell, thereby shielding them from rapid enzymatic degradation and increasing their surface-area-to-volume ratio. This significantly improves their dissolution rate and water solubility, facilitating better passive diffusion across the intestinal epithelium and ensuring that therapeutic concentrations reach the target tissues more effectively [18,20]. Tetrahydrocurcumin is also presented as a structural CUR analogue [11,12]. RSV further possesses dose-dependent antitumor effects against gastric cancer. At low concentration (100 μM), it was observed to enhance cell viability. On the other hand, at high concentration (1000 μM), it caused cytotoxicity through the generation of ROS. Cisplatin also contributed to ROS generation. Highest production was observed when they were administered in combination [54]. The use of super porous hydrogels is highly promising for the delivery of hydrophobic anticancer agents. More specifically, SPHs address the low bioavailability of orally administered RSV (3.2 times higher relative bioavailability). This is achieved through their prolonged residence in the stomach for a longer period than is usually observed (12 h compared to <2 h). Controlled drug delivery systems such as nanoparticles and superporous hydrogels have been shown to reduce systemic toxicity by enabling sustained release and targeted action. Issarachot et al. [20] demonstrated an enhancement of anticancer activity, with the IC50 value reduced to 25 μM compared to 50 μM in vitro; however, further validation in clinical settings is required. In this way, they also indicate their therapeutic potential in stomach cancer. It is important that future studies focus on the clinical documentation of these compounds. Refinement of better combination methods and new formulations for delivery is needed. Despite the promising preclinical data, the transition to clinical practice faces critical limitations in trial designs, such as small sample sizes, lack of standardized dosing protocols, and short follow-up periods [8,16]. Furthermore, patient-related factors, including variations in gut microbiota and genetic polymorphisms in metabolic enzymes, are often overlooked, leading to significant inter-individual variability in drug response [32]. The clinical translation of scientific findings from the laboratory to their application in patients is accompanied by significant challenges. Initially, one of the main obstacles is the limited reproducibility of preclinical results in humans, because cell or animal models do not fully reflect human pathophysiology [23]. The biological heterogeneity of patients, combined with an incomplete understanding of molecular mechanisms of action, further complicates efficacy and safety [22]. In addition, issues of safety, toxicity, regulatory requirements and difficulties in mass production constitute serious obstacles [18]. In order to address these challenges, the development of more reliable and human-centered preclinical models and the implementation of rigorous, standardized methodologies are proposed [21]. The timely assessment of pharmacokinetic parameters, toxicity and biomarkers, along with appropriate selection and stratification of patients, can improve the effectiveness of clinical studies [41,48]. Finally, close collaboration between basic investigators, clinicians, and regulatory agencies, alongside early planning for regulatory approval and scale-up of production, is crucial for successful clinical application [54,55]. In addition, development of pharmacokinetics could be necessary to fill the gap between preclinical and clinical application for gastric cancer therapy [18,57]. Recent studies suggest that hybrid molecules combining structural features of CUR and RSV may enhance their antitumor activity and selectivity [9,54]. Advanced targeting strategies, such as ligand-functionalized nanoparticles, superporous hydrogels, or encapsulation methods, could further improve bioavailability and tissue-specific delivery [18,20]. Finally, situating this review in the context of prior studies clarifies its focus on preclinical and delivery strategy advancements, highlighting gaps for future investigation [9,29].

## 5. Conclusions

Curcumin (CUR) and resveratrol (RSV) have been recognized as two active natural polyphenols with particularly important therapeutic functions during the treatment of gastric cancer (GC). Both exhibit extremely important biological features. This renders them to serve as supplement medicines in modern cancer treatments. Their synergistic effects, combined with existing chemotherapeutic drugs, are also important. Therefore, they enhance the effectiveness of treatment and overcome therapeutic resistance mechanisms. However, there are major limitations even today, despite the recent advances in drug delivery technology aimed at providing solid solutions for optimal clinical usage. Future research should prioritize the development of more efficient and clinically viable drug delivery systems to overcome critical barriers such as poor water solubility, rapid metabolism, and low bioavailability of CUR and RSV. Approaches such as nanoparticle encapsulation, liposomes, and superporous hydrogels have already shown promise in preclinical models and warrant further optimization. Additionally, rigorous and large-scale clinical trials are essential to establish the therapeutic efficacy, safety, and optimal dosing of these compounds, both individually and in combination with conventional chemotherapy regimens. Dose-dependent effects and potential toxicity at high concentrations should be carefully evaluated. Integrating these agents into clinical oncology requires addressing several key challenges, including pharmacokinetics, delivery route selection, and interpatient variability. Tailored formulations based on predictive biomarkers, such as lncRNA profiles (e.g., MALAT1) or ROS sensitivity, may allow for personalized therapy in the future.

## Figures and Tables

**Table 1:** Differential targeting of oncogenic pathways by CUR and RES, including apoptosis regulators, cell cycle checkpoints, EMT/metastasis suppressors, and chemosensitization markers.

Category	Curcumin (CUR)	Resveratrol (RSV)
**Apoptosis**	Activation of caspases-3, -8, -9	Activation of p53, p21, p16
	PARP cleavage	Increased ROS
	Bax increase/Bcl-2 decrease	Inhibition of EMT
	ER stress (CHOP, JNK)	Cellular senescence via β-Gal
**Oxidative stress/OR**	ROS increase	Increased ROS in combination with DDP
	Dual role (Nrf2/HO-1 activation & ferroptosis)	Antioxidant properties via SOD, CAT, GPX
**Cell Cycle**	Arrest in G0/G1 & G2/M	Arrest in G0/G1 & G2/M
	Cyclin D1, CDK4/6 suppression	Increased p21, p53
**Antiproliferative effects**	Wnt3a, LRP6, β-catenin inhibition	Suppression of MALAT1
	c-Myc, survivin reduction	Inhibition of cell migration and invasion
**Pathway** **PI3K/Akt/mTOR**	Akt and mTOR inhibition	Activation of PTEN
	PTEN enhancement via miR-21 inhibition	Suppression of EMT
		Inhibition of Akt
**Modulation of miRNA/lncRNA**	↑ miR-34a → ↓ CDK4, Cyclin D1, Bcl-2	↓ MALAT1
	↓ miR-21 → ↑ PTEN	Combination with si-MALAT1 increases apoptosis
	LINC01021, AC022424 regulation	
**Anti-metastatic effects**	EMT inhibition	Inhibition of EMT
	↑ E-cadherin	↓ MMP-2
	↓ Snail, Vimentin	Inhibition of cell migration
	↓ MMP-9/↑ MMP-2	
**Anti-angiogenesis**	↓ VEGF, LYVE-1, VEGFR-3	Reduction in inflammatory cytokines (IL-6, TNF-α) via NF-κB suppression
**Anti-inflammatory effects**	Through NF-κB inhibition	NF-κB inhibition
		↓ IL-1β, IL-6, TNF-α
**Interaction with Chemotherapy**	Combination with 5-FU, DOX, VCR, Cisplatin increases efficacy	Combination with DDP, DOX
	↓ MDR1, MRP1	Reduction in resistance via PTEN
		↓ Cu toxicity
**New forms of administration**	Photodynamic therapy (PDT)	RES@ZIF-90 nanoparticles with enhanced anticancer effect
	Combinations with FOLFOX	
**Anti-Helicobacter pylori**	Inhibition of urease, neutralization of VacA, ↑ antioxidant capacity	-
**Ferroptosis**	↑ ACSL4, ↓ SLC7A11, GPX4 → induction of iron-dependent apoptosis	-

## Data Availability

No new data were created or analyzed in this study. Data sharing is not applicable to this article.

## Abbreviations

The following abbreviations are used in this manuscript:

5-FU	5-Fluorouracil
AGS	Human gastric adenocarcinoma cell line
Bax	Bcl-2-associated X protein
Bcl-xl	B-cell lymphoma-extra large
CAT	Catalase
CagA	Cytotoxin-associated gene A (H. pylori virulence factor)
CUR	Curcumin
DDP	Cisplatin
Des1/Des2	Dihydroceramide desaturases 1 and 2
DOX	Doxorubicin
EMT	Epithelial–mesenchymal transition
ER	Endoplasmic reticulum
GC	Gastric cancer
GPX	Glutathione peroxidase
LVD	Lymphatic vessel density
MDA	Malondialdehyde
MDR	Multi-drug resistance
NF-κB	Nuclear factor kappa B
NRf2	Nuclear factor erythroid 2-related factor 2
PKC	Protein kinase C
PS	Photosensitive photosensitizer
ROS	Reactive oxygen species
RSV	Resveratrol
SOD	Superoxide dismutase
SPHs	Sphingolipids (context-specific abbreviation; e.g., sphingosine species)
TCF	Transcription factor
VCR	Vincristine
